# Update of the helminth fauna in Eurasian lynx (*Lynx lynx*) in Poland

**DOI:** 10.1007/s00436-018-5953-0

**Published:** 2018-06-14

**Authors:** Marta Kołodziej-Sobocińska, Yegor Yakovlev, Krzysztof Schmidt, Zuzana Hurníková, Iwona Ruczyńska, Michał Bednarski, Małgorzata Tokarska

**Affiliations:** 1 Mammal Research Institute, Polish Academy of Sciences, Stoczek 1, 17-230 Białowieża, Poland; 2 Schmalhausen Institute of Zoology NAS of Ukraine, Khmelnytskogo, 15, Kyiv, 01601 Ukraine; 3 Kyiv Zoological Park of National Importance, Prospect Peremohy, 32, Kyiv, 04116 Ukraine; 4 Institute of Parasitology, Slovak Academy of Sciences, Hlinkova 3, 040 01 Košice, Slovak Republic; 5 Department of Epizootiology and Clinic of Bird and Exotic Animals, Wrocław University of Environmental and Life Sciences, Pl. Grunwaldzki 45, 50-366 Wrocław, Poland

**Keywords:** Carnivores, Felidae, Eurasian lynx, Helminth fauna, Endangered species

## Abstract

The Eurasian lynx (*Lynx lynx*) is a strictly protected species of large carnivore in Poland. It inhabits forest complexes in north-eastern Poland and the Carpathian region in southern Poland. The status of the lynx within Poland requires special attention because its range decreased between 1980 and 2001 and has not yet recovered. One of the factors negatively affecting lynx populations is diseases, particularly parasites. The helminth fauna of the Eurasian lynx is not fully known in Poland. Previous coprological studies revealed that Polish lynx have been infected with seven species of nematodes, three species of cestodes, and one species of trematode. In this study, we present new data based on examination of opportunistically collected lynx carcasses. The aim of the study was to complement data on the helminth fauna of Eurasian lynx inhabiting Poland based on morphological and molecular analysis of parasites. Four species of cestodes—*Taenia lynciscapreoli*, *Mesocestoides lineatus*, *Spirometra* sp., and *Taenia krabbei*—were found for the first time in Eurasian lynx from Poland and three previously reported species of nematodes—*Ancylostoma tubaeforme*, *Toxascaris leonina*, and *Toxocara cati*—were confirmed. Larvae of *Trichinella britovi* were also detected in Eurasian lynx in Poland for the first time.

## Introduction

The Eurasian lynx (*Lynx lynx*) is a strictly protected large carnivore species in Poland. Despite protection and a general increasing trend in other countries (Chapron et al. 2014), its population within Poland decreased dramatically between 1980 and 2001 (Jędrzejewski et al. 2002) and has not yet recovered. Habitat fragmentation in central Europe has been suggested as a major cause of this situation due to a strong affiliation between these felids and forest habitat (Kowalczyk et al. 2015; Podgórski et al. 2008; Schmidt 1998). Infestation with parasites has been shown to be an important mortality factor in lynx (Breitenmoser et al. 2000; Schmidt-Posthaus et al. 2002; Andren et al. 2006; Szczęsna et al. 2008). Therefore, monitoring parasite load in wildlife showing negative population trends may be crucial for understanding their status.

The helminth fauna of Eurasian lynx in Europe reported so far consists of 25 species including 11 species of cestodes, one species of trematode, and 14 species of nematodes (Furmaga 1953; Merkusheva and Bobkova 1981; Yushkov 1995; Samuel et al. 2001; Bagrade et al. 2003; Valdmann et al. 2004; Szczęsna et al. 2008; Kornnyushin and Varodi 2010; Deksne et al. 2013; Lavikainen et al. 2013; Haukisalmi et al. 2016). Data from lynx in Poland are limited due to rarity of this species and are based on either scant material or fecal samples, which provide underestimated results of helminth prevalence and intensity (Szczęsna et al. 2008). The presence of parasites such as *Diphyllobothrium latum* (L., 1758); *Hydatigera* (=*Taenia*) *rileyi* (Loewen, 1929) (misidentification, *Taenia* sp. (Fagasiński, 1961) according to Abuladze [18]); *Spirometra janickii* Furmaga, 1953; *Alaria alata* (Goeze, 1782); *Aelurostrongylus abstrusus* (Railliet, 1898); *Ancylostoma tubaeforme* (Zeder, 1800); *Eucoleus* (=*Thominx*) *aerophilus* (Creplin, 1839); *Metastrongylus* sp.; *Nematodirus* sp.; *Toxocara mystax* (=*T. cati*) (Schrank, 1788) Baylis et Daubney, 1923; and *Toxascaris leonina* (Linstow, 1902) was confirmed to date (Furmaga 1953; Fagasiński 1961; Okulewicz et al. 2002; Górski et al. 2006; Szczęsna et al. 2006, 2008; Filip and Demiaszkiewicz 2017).

In this study, we present new data based on examination of opportunistically collected lynx carcasses. The aim of the study was to contribute data on the helminth fauna of Eurasian lynx inhabiting Poland based on morphological and molecular analysis of parasites. Due to the protected status of lynx in Poland, such comprehensive analyses give a unique opportunity to increase the knowledge on potential limiting factors for the sustainability of the fragmented population of this large felid.

## Material and methods

### Animal dissections and collecting of helminths

**Fig. 1:**
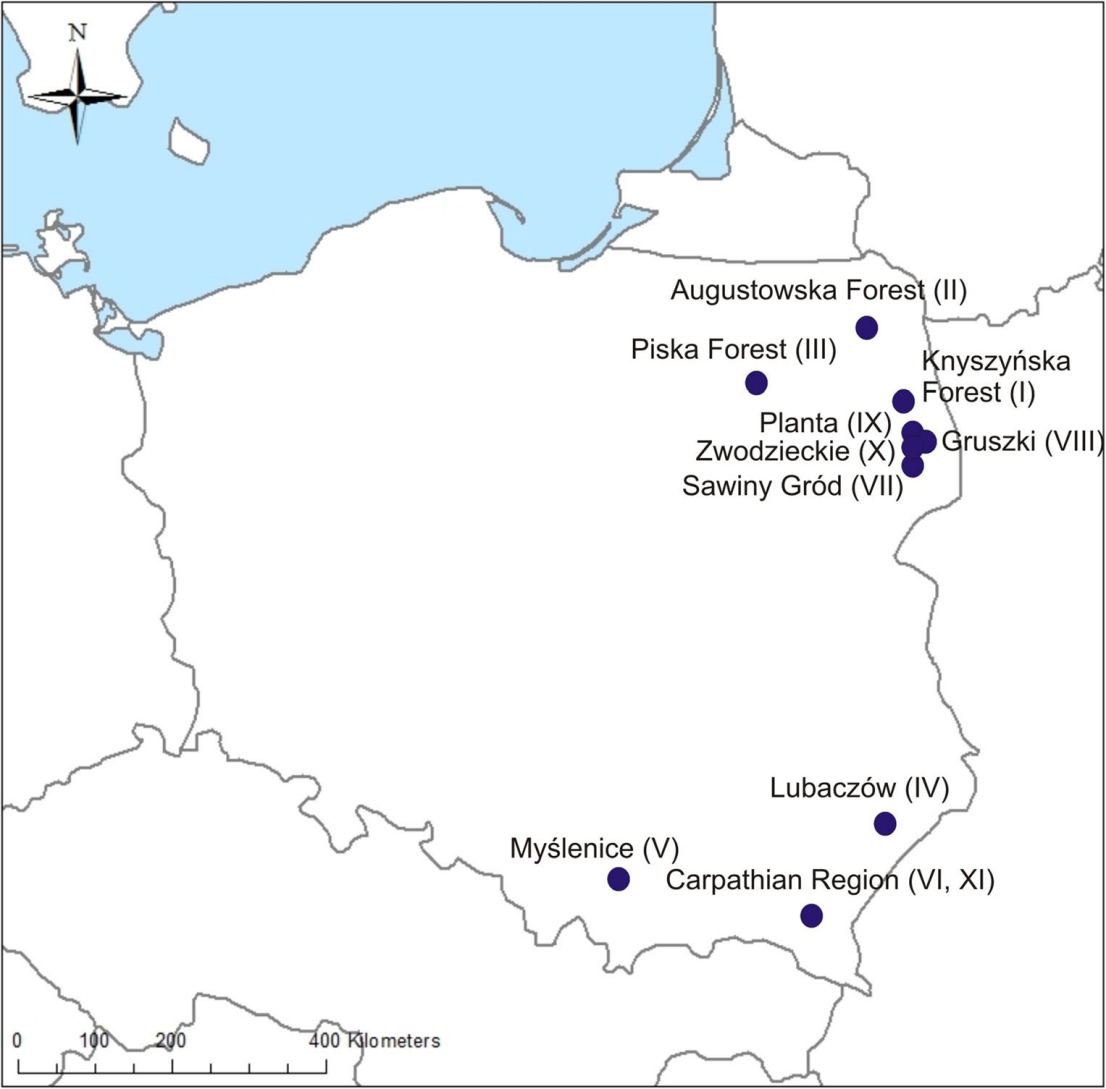
An outline map of Poland showing sample collection locations. Roman numerals in brackets represent ID numbers of studied lynx

Eleven lynx carcasses were collected in 2007–2017 in northern-eastern and southern Poland (Fig. 1). In all cases, except one which was a road fatality, the lynx were found dead and some of them were strongly emaciated (Table 1). Gastrointestinal tracts and muscle samples were collected for parasitological studies. Isolated helminths were preserved in 70% ethanol for further analysis.

**Table 1:** Details of the Eurasian lynx studied for helminth fauna and the helminths found in each individual. NE, north-eastern Poland; S, southern Poland; LPG, number of *Trichinella* larvae per gram of muscle for positive animals. For other parasite species, the number of individuals is presented. Juveniles, up to 1 year; subadults, > 1–2 years; adults, > 2 years

Lynx no.	Localization	Date of death	Circumstances of death	Age	Sex	Parasites	Number of cestodes	Number of nematodes
I	Knyszyńska Forest (NE)	12.04.2010	Found dead, cause unknown	Juvenile	Female	*Taenia lynciscapreoli*, *Taenia krabbei*, *Toxocara cati*	50	2
II	Augustowska Forest (NE)	12.06.2009	Killed by car	Adult	Male	*Taenia lynciscapreoli*, *Trichinella britovi*	53	3.2 LPG
III	Piska Forest (NE)	17.07.2009	Found dying, highly emaciated in a dog kennel	Subadult	Female	*Taenia lynciscapreoli*, *Ancylostoma tubaeforme*, *Toxocara cati*	8	62
IV	Lubaczów (S)	10.02.2013	Found dead, autopsy suggested poisoning by unknown substance	?	Male	*Mesocestoides lineatus*, *Spirometra* sp., *Taenia lynciscapreoli*, *Taenia krabbei*, *Ancylostoma tubaeforme*, *Toxocara cati*	180	90
V	Myślenice (S)	01.07.2007	Found dead, strongly emaciated body, together with two siblings	Juvenile	Female	*Taenia krabbei*, *Toxocara cati*	1	1
VI	Carpathian Region (S)	02.09.2010	Found dying, strongly emaciated, death caused by prenatal pathology	Subadult	Female	*Taenia lynciscapreoli*	7	0
VII	Białowieża Forest (NE)	25.11.2015	Found dead, highly emaciated in farm buildings	Juvenile	Male	*Taenia krabbei*, *Toxocara cati*	11	1
VIII	Białowieża Forest (NE)	28.02.2016	Found dying, highly emaciated, sarcoptic mange infection	Adult	Female	*Taenia lynciscapreoli*, *Toxocara cati*, *Trichinella britovi*	45	3, 1.8 LPG
IX	Białowieża Forest (NE)	28.02.2016	Found dead near the village, sarcoptic mange infection	Juvenile	Female	*Taenia lynciscapreoli*, *Toxocara cati*	32	17
X	Białowieża Forest (NE)	02.01.2017	Found dead, probably bitten by another lynx	Adult	Female	*Taenia lynciscapreoli*, *Trichinella* sp.	24	0.1 LPG
XI	Carpathian Region (S)	19.04.2017	Found dead, sarcoptic mange infection	Adult	Female	*Teania krabbei*, *Toxascaris leonina*	8	9

### Morphological identification of parasites

For light microscope observation, nematodes were processed in 80% solution of phenol in glycerol, for measurement nematodes were processed in enlightening solution made from lactic acid, phenol, glycerol, and distilled water (1:1:1:1). Cestode scolexes were processed in a 50% solution of glycerol in distilled water. The total amounts of scolexes were kept in Berleses medium, and mature and immature proglottids were stained with iron acetocarmine and mounted in Canada balsam (Ivashkin et al. 1971). Observations were done using Amplival Carl Zeiss Jena and Zeiss Axio Imager M1 microscopes and Leica M165C and MBS-9 stereomicroscopes.

Species identification of parasites was based on morphological features. In the case of damaged specimens, a standard coproscopic parasite eggs identification was additionally performed. Comparison with the collection of helminths from the Department of Parasitology of the Schmalhausen Institute of Zoology, specific keys to parasitic nematodes, Mesocestoidata, and Taeniata was used (e.g., Skryabin et al. 1951, 1952; Mozgovoy 1953; Abuladze 1964; Ryzhikov 1978). Infection of Eurasian lynx with helminths was presented by standard parameters: prevalence—number of infected hosts divided by the total number of dissected animals (shown in the form of simple fraction); mean intensity—the total number of parasites divided by the number of infected lynx; mean abundance—the total number of parasites divided by the total number of analyzed animals (Bush et al. 1997).

### Digestion and molecular identification of *Trichinella* species

Muscle samples (diaphragm or foreleg muscle) from eight lynx (15 g each) were individually examined for the presence of *Trichinella* muscle larvae using the magnetic stirrer method for sample digestion as described by Kapel and Gamble (2000). Obtained muscle larvae were identified based on morphological characteristics and counted, and the number of larvae per gram of muscle tissue (LPG) was calculated. Larvae were washed several times in distilled water and stored in 96% ethanol until speciation by the multiplex polymerase chain reaction (PCR).

DNA was isolated from a pool of five larvae from each sample (if available). Extraction of DNA and PCR amplification was performed according to Pozio and La Rosa (2003) modified by using two pairs of primers: ESVF (5′-GTTCCATGGAACAGCAGT) and ESVR (5′-CGAAAACATACGACAACTGC) amplifying the expansion segment V (ESV) region in all known *Trichinella* species and genotypes and TBF (5′-GCTACATCCTTTTGATCGGTT) and TBR (5′-AGACACAATATCAACCACAGTACA) that amplify a partial region of ITS-1 specific for *T. britovi* Britov et Boev, 1972. For amplification, 5 μl DNA was added to the PCR reaction mix (vol. 25 l) by Eppendorf® and PCR was performed in a XP ThermalCycler (BIOER Technology Co., LTD.). Amplification was carried out under the following cycling conditions: 40 cycles of denaturation at 94 °C for 30 s, annealing at 58 °C for 30 s, and elongation at 72 °C for 1 min. The amplification products (5 μl) were separated on a 1.6% standard agarose gel and stained with GoldView™ Nucleic Acid Stain before visualization. All PCRs included negative and positive controls—reference strains *T. spiralis* Owen, 1835 (ISS 004); *T. nativa* Britov et Boev, 1972 (ISS 042); *T. britovi* (ISS 1088); and *T. pseudospiralis* Garkavi, 1972 (ISS 013).

### Genetic identification and phylogenetic analysis of *Taenia* species

To verify the results of microscope examination of the *Taenia* species that could be misidentified by morphological features, we applied three primer pairs, constructed by Jia et al. (2010), designed for purposes of comparative mitogenomics of *Taenia* species. The three primer pairs enabled the amplification of four distinct mtDNA fragments containing cytochrome c oxidase (cox, approx. 470 bp), nicotinamide dehydrogenase subunits 1 (nad1, approx. 460 bp) and 5 (nad5, approx. 600 bp), and the small unit of rRNA (rrnS, approx. 400 bp). Amplifications of the fragments were performed in 10 μl of reaction mixture, including 2 μl of the DNA, 5 μl of PCR HotStarTaq Master Mix Kit, and 1 μl of each of two primers (5 pmol/μl). Thermal cycling conditions: 35 cycles of denaturation at 95 °C for 60 s, annealing at 58 °C for 45 s (T_nad1 and T_nad5), 65 °C for 45 s (T-rrnS), and 54 °C for 60 s (Cox1), and elongation at 72 °C for 10–15 min.

To increase the possibility to detect all possible *Taenia* species, we sampled 1–4 distinct parasites from each lynx, depending on their individual availability. The obtained sequences were BLASTed using the NCBI online tool (http://blast.ncbi.nlm.nih.gov/Blast.cgi) (Altschul et al. 1990). Sequence alignments between the obtained sequences and the evolutionarily closest sequences were performed using ClustalW Multiple Alignment option delivered within BioEdit tools (Hall 1999). Molecular phylogenetic analysis of the lynx parasites by Maximum Likelihood method based on the Tamura-Nei model within MEGA6 tool (Tamura and Nei 1993; Tamura and Kumar 2004) was performed for all the samples and all three mtDNA fragments.

Mean genetic distances between taxa (average evolutionary divergence over sequence pairs between groups of sequences) and within taxa, expressed as the number of base substitutions per site from averaging over all sequence pairs between groups, were estimated based on maximum composite likelihood model (Tamura and Nei 1993) using MEGA6 (Tamura et al. 2013).

## Results

All lynx we examined were infected with helminths. We found eight species of parasites, consisting of four species of cestodes: *Mesocestoides lineatus*, *Spirometra* sp., *Taenia krabbei*, and *Taenia lynciscapreoli* and four species of nematodes: *Ancylostoma tubaeforme*, *Toxocara cati*, *Toxascaris leonina*, and *Trichinella britovi* (Tables 1 and 2). The four cestode species mentioned above and one nematode (*T. britovi*) are reported for the first time in lynx inhabiting Poland.

**Table 2:** Parasites of Eurasian lynx found in Poland. Species of parasites identified for the first time in lynx from Poland are in bold. Method: D, dissection; F, fecal analysis. Localization in Poland: E, eastern; NE, north-eastern; S, southern; NW, north-western; SW, south-western

Parasite	Localization in Poland	Method	References
**Nematoda**			
*Angiostrongylus abstrusus*	E	F	Szczęsna et al. 2006, 2008
*Ancylostoma tubaeforme*	E, NE, S	F, D	Szczęsna et al. 2008 This study
*Eucoleus aerophilus*	E	F	Szczęsna et al. 2008
*Metastrongylus* sp.	E	F	Szczęsna et al. 2008
*Nematodirus* sp.	E	F	Szczęsna et al. 2008
*Toxocara cati*	E, NE, S	F, D	Fagasiński 1961; Górski et al. 2006; Szczęsna et al. 2008 This study
*Toxascaris leonina*	NW, SW	F	Okulewicz et al. 2002; Filip and Demiaszkiewicz 2017 This study
***Trichinella britovi***	NE	D	**This study**
**Cestoda**			
*Diphyllobothrium latum*	E	F	Szczęsna et al. 2008
***Mesocestoides lineatus***	S	D	**This study**
*Spirometra janickii*	E	F, D	Furmaga 1953; Szczęsna et al. 2008
***Spirometra*** **sp.**	E	D	**This study**
***Taenia krabbei***	NE, S	D	**This study**
***Taenia lynciscapreoli***	NE, S	D	**This study**
*Taenia* sp.	NE	D	Fagasiński 1961
**Trematoda**			
*Alaria alata*	E	F	Szczęsna et al. 2008

**Fig. 2:**
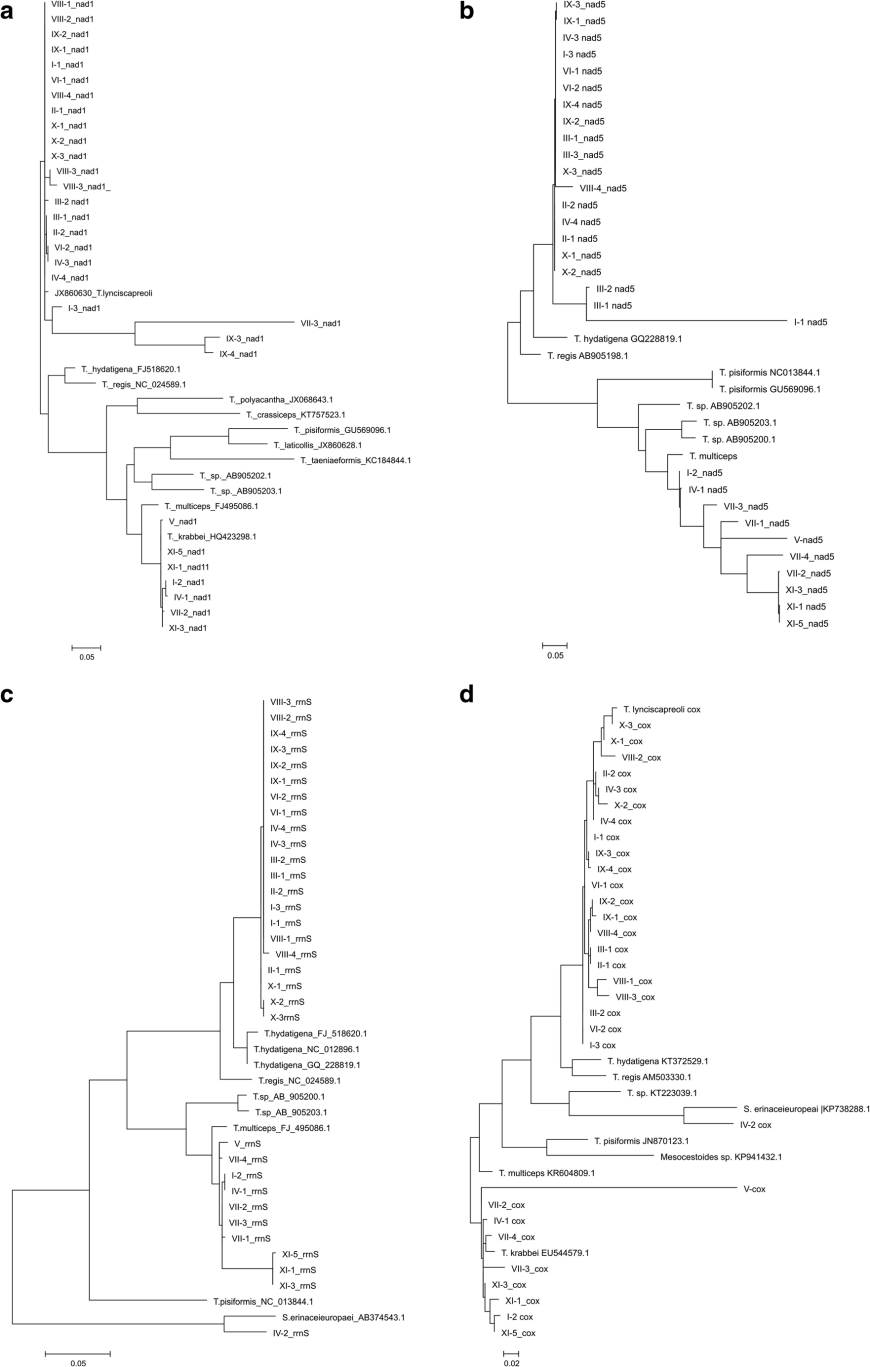
Phylograms for the four mtDNA fragments of the lynx parasite DNA samples collected in the study (in bold type) and the reference sequences from GeneBank (National Center for Biotechnology Information, http://www.ncbi.nlm.nih.gov/). Evolutionary relationships of the taxa were implied using the Neighbor-Joining method (Tamura and Nei 1993) embedded in MEGA6 software (Tamura et al. 2013). The evolutionary distances were computed using the maximum composite likelihood method (Tamura and Kumar 2004) and are expressed in units of the number of base substitutions per site. The molecular phylogenies were drawn based on the following mtDNA fragments: **a** nicotinamide dehydrogenase subunit 1 (nad1); **b** nicotinamide dehydrogenase 5 (nad5), **c** small unit of rRNA (rrnS); and **d** cytochrome c oxydase (cox). Roman numerals indicate lyx ID, while Arabic numerals preceded by a dash indicate subsequent sampled parasite individuals

Most prevalent was *T. lynciscapreoli*, found in 8 out of 11 studied lynx. We found *Taenia krabbei* in five lynx and both *M. lineatus* and *Spirometra* sp. in one of the lynx. Three individuals were infected with *Trichinella* sp. with intensity of the latter at 0.1 to 3.2 LPG. *T. britovi* was confirmed by PCR in two lynx. The highest infection intensity was observed for *M. lineatus* and the lowest for *Spirometra* sp. (see Table 3). We observed an untypical location of *Spirometra* sp., in the large intestine. The results of interspecific mtDNA alignment are presented in Fig. 2a–d.

**Table 3:** Descriptive statistics for helminth infections in Eurasian lynx in Poland

Parasites	Prevalence	Mean intensity	Mean abundance
**Nematoda**	**9/11**	**39**	**32**
*Toxocara cati*	7/11	26	17
*Toxascaris leonina*	1/11	9	1
*Trichinella britovi*	3/8	–	–
*Ancylostoma tubaeforme*	2/11	26	5
**Cestoda**	**6/11**	**50**	**27**
*Mesocestoides lineatus*	1/11	139	13
*Spirometra* sp.	1/11	6	1
*Taenia krabbei*	5/11	6	3
*Taenia lynciscapreoli*	8/11	24	17

The aggregate results of detected in lynx Nematoda and Cestoda parasites are in bold

## Discussion

Our study provided new information on the helminth species of lynx in the territory of Poland. Until now, the accumulated data from the literature along with the results of this study show that the lynx in this area can be a host to at least 14 species of helminths. This data is important considering that lynx populations in Poland are unstable or decreasing (Von Arx et al. 2004), and in central Europe, they are also strongly fragmented and limited to forest areas (Kowalczyk et al. 2015; Schmidt 1998). Therefore, every risk that may contribute to deepening the vulnerability of the population, including parasitic invasions, must be taken into account in conservation strategies.

Lynx feed on ungulates (~ 90% of prey) with roe deer (*Capreolus capreolus*) and red deer (*Cervus elaphus*) being the main prey species (Okarma et al. 1997), with smaller animals being preyed upon only occasionally. Intermediate hosts for *T. lynciscapreoli* and *T. krabbei* Moniez, 1789, are roe deer and other cervids (Al-Sabi et al. 2013; Haukisalmi et al. 2016). Thus, the relatively high *Taenia* sp. infection prevalence stated in our study is directly connected with lynx feeding habits. According to our best knowledge, this is the first reported case of lynx as the definitive host for *T. krabbei* that usually uses canids as its definitive hosts (Priemer et al. 2002; Lavikainen et al. 2011). The life cycle of *M. lineatus* has not yet been clarified. However, the first intermediate host could be oribatid mites, and the range of the second intermediate hosts is quite wide, including species from different classes of vertebrates (Ryzhikov 1978; Yushkov 1995), which could form part of the lynx diet. *T. cati* and *A. tubaeforme* are widespread helminths of cats with *A. tubaeforme* being a specific parasite of felids. Both species infect hosts percutaneously in dens, during feeding (engulfment of eggs or larvae in rodents or insects or with milk) or through the placental barrier. However, no *larvae migrans* of these nematodes were found in our study.

Our data suggest that the confirmed southern range of *T. lynciscapreoli* should be extended. Up to-date, the species has been found and reported in Lohja, Hyvinkää, Hausjärvi (Southern Finland), Mustasaari, Sauvo (Western Finland), Mikhailovskiy raion (Altai Krai), Kolosovsky, and Bolsheukovsky raions (Omskaya oblast, Western Siberia) in the Russian Federation (Haukisalmi et al. 2016).

To our knowledge, we report the first finding of a *Trichinella* parasite in Eurasian lynx from Poland. *T. britovi* was detected in two adult females from Białowieża Forest and an adult male from Augustów Forest. The presence of *Trichinella* in lynx is not surprising, as the species is known to feed occasionally on carnivorous and scavenging mammals (Okarma et al. 1997). Trichinellosis in Eurasian lynx was found in 22 to 67% of individuals in Estonia (Järvis and Miller 1999; Valdmann et al. 2004; Malakauskas et al. 2007; Pozio 2013) in 46 to 98% in Republic of Latvia (Bagrade et al. 2003; Malakauskas et al. 2007) and in Finland, where the prevalence in lynx is the highest among free-living carnivores—up to 70% (Airas et al. 2010). *T. britovi* was detected also in lynx from Slovakia (Hurníková et al. 2009) and in 27% of Swiss lynx (Frey et al. 2009). This species is also prevalent in other wild mammals in Poland, infecting mainly red fox (Cabaj et al. 2000; Cabaj et al. 2004), but also found in wild boar (*Sus scrofa* L., 1758) (Bilska-Zając et al. 2013), wolf (Cabaj 2006), martens and badger (Moskwa et al. 2012), and American mink (*Neovison vison* Schreber, 1777) (Hurníková et al. 2016).

The number of dissected animals in this study was too small to derive conclusions about possible relationships between parasite infestation intensity and lynx mortality or morbidity. However, in the majority of cases, the dead lynx were found emaciated and without an apparent cause of death. However, the harmful influence of helminth invasion on animal health status has been shown previously (Rodriguez and Carbonell 1998).

This study conducted on the rare and unique material of dead lynx from the territory of Poland has yielded unique knowledge of lynx helmith fauna. All these findings may be helpful in explaining parasite transmission patterns in the areas inhabited by lynx. Five new pathogenic agents discovered in endangered Eurasian lynx on the Polish territory could have a negative impact on its condition and fitness.
